# The Relationship Between Spiritual Care Needs and Death Anxiety in Turkish Patients with Chronic Obstructive Pulmonary Disease

**DOI:** 10.1007/s10943-025-02284-9

**Published:** 2025-03-14

**Authors:** Ezgi Yıldız, Feride Taskin Yilmaz, Şerife Karagözoğlu

**Affiliations:** 1 Susehri School of Health Nursing Department, Sivas Cumhuriyet University, Sivas, Turkey; 2 Faculty of Health Sciences, Sakarya University of Applied Sciences, Sakarya, Turkey; 3 Faculty of Health Sciences Fundamentals of Nursing Department, Sivas Cumhuriyet University, Sivas, Turkey

**Keywords:** COPD, Death anxiety, Spirituality, Need for spiritual care

## Abstract

This study aimed to determine the levels of spiritual care needs and death anxiety, as well as the relationship between them, in Turkish patients with chronic obstructive pulmonary disease (COPD). This descriptive and correlational study included 480 patients who were admitted to the pulmonology outpatient clinics of a public hospital with a diagnosis of COPD between November 1, 2023, and April 31, 2024. The study data were collected using the Patient Diagnosis Form, Dyspnea Fear Level Assessment Form, Spiritual Care Needs Inventory, and Templer Death Anxiety Scale. It was determined that 60.8% of the patients had high death anxiety and moderate spiritual care needs. A positive correlation (r = 0.327; *p* < 0.01) was found between the mean scores of the Spiritual Care Needs Inventory and the Templer Death Anxiety Scale. Patients with high death anxiety also had high spiritual care needs (*p* < 0.01). It was determined that the gender of the patients, fear of dyspnea, and death anxiety predicted 20% of the need for spiritual care (R = 0.457, R^2^ = 0.209, F = 17.800, *p* < 0.001). Considering the study findings, it can be stated that death anxiety may decrease as the spiritual needs of the patients are met. Within the framework of holistic care, it is essential for health professionals to identify patients’ spiritual needs in addition to their physical and psychological needs and to implement interventions to meet these needs.

## Introduction

Chronic obstructive pulmonary disease (COPD) is an irreversible pathological condition in which airflow is limited. Airflow limitation is usually progressive and is associated with an abnormal pulmonary inflammatory response (da Silva et al., 2009). COPD is one of the diseases with a social and economic burden due to its morbidity and mortality rates worldwide (Venkatesan, 2024). Worldwide, the overall prevalence of COPD in individuals aged 40 years and older is 12.64% (Al Wachami et al., 2024). In Turkey, COPD is the fourth leading cause of death after ischemic heart disease, stroke, and lung cancer (Turkey Noncommunicable Diseases & Risk Factors Cohort Study, 2021).

Dyspnea, cough, expectoration, and acute exacerbations are symptoms specific to COPD (Venkatesan, 2024). Dyspnea, which has been gradually increasing over the years, initially occurs with exertion but later reaches levels that interfere with activities of daily living and affect self-care abilities (Demir Gokmen & Firat, 2022; Tuluce et al., 2016). Moreover, psychosocial problems, such as future anxiety and hopelessness, may occur in patients with COPD due to continuous medication use, hospital dependency, and dysfunction of affected organs (Togluk & Cuhadar, 2021). These physical and psychological limitations experienced by patients with COPD may cause death anxiety (Demir Gokmen & Firat, 2022; Tuluce et al., 2016; Turhal et al., 2021). Death anxiety can be defined as the anxiety that individuals feel in the face of the fact that their existence in the world will come to an end. Death anxiety is a cornerstone of individual fears and anxieties (Togluk & Cuhadar, 2021). Excessive anxiety regarding death can complicate individuals’ balance and adaptation in daily life (Karakuş et al., 2012).

Studies examining death anxiety in patients with COPD are limited (Demir Gokmen & Firat, 2022; Nal et al., 2016; Okur & Nural, 2022; Togluk & Cuhadar, 2021). One of these studies determined that as the severity of dyspnea increased, death anxiety intensified with the fear of not being able to breathe (Okur & Nural, 2022). In another study, it was reported that dyspnea-induced intolerance to physical activity, coughing frequency, family dependency, anxiety, and depression increased the sense of nearness to death (Silva et al., 2009). Planning supportive and preventative health measures for COPD patients is thought to depend heavily on their level of death anxiety, and further research is required in this area.

Building up a person’s spiritual domain and giving them the necessary spiritual care is one strategy suggested in the literature to deal with death anxiety (Cinar & Aslan, 2017; Fradelos et al., 2021). Spirituality is the individual’s effort to comprehend and accept their relationship with themselves and others, their place in the universe, and the meaning of life (Gergianaki et al., 2019; Kacal & Demirsoy, 2018). All individuals have a spiritual dimension that they have always had from birth. Individual’s spiritual lives are just as vital as their physical, emotional, and social lives (Cinar & Arslan, 2017; Ismailoglu et al., 2019; Yousefi & Abedi, 2011). When looking at the relationship between spirituality and health, it is generally seen that spiritual practices prevent bad habits and naturally lead people to live a healthy life, eat healthy, and live a planned life. In addition, it is stated in the literature that patients with a developed spiritual side are healthier physically, emotionally, and socially (Boztilki & Ardic, 2017).

In times of crisis, when people are unwell, stressed, afraid of dying, doubt life’s purpose, and lose faith, the spiritual side becomes more apparent. In particular, life-threatening illnesses lead to the emergence of spiritual care needs (Fradelos et al., 2021). Spiritual needs are those that contribute to the spiritual development of the individual and make the person a sociable, hopeful individual who is always grateful to God (Yousefi & Abedi, 2011). Individuals’ spiritual needs are met through human relationships or a connection with God. In this regard, spiritual care is defined as spiritual support services provided to patients who request it, provided that it does not adversely affect their medical treatment, to provide spiritual advice, to support them spiritually and morally, to guide them to fulfill their prayers as much as their illness allows, and encouraging them to hold onto their hope for a better life (Cinar & Arslan, 2017).

Nursing care for the spiritual needs of patients can be provided by practices such as showing compassion by empathizing and helping patients realize their existence, evaluating all physical, emotional, and spiritual aspects of patients concerning each other, learning patients’ spiritual backgrounds, evaluating the symptoms of spiritual concerns/anxieties, listening to patients’ fears, hopes, pain, and dreams, accepting their words without prejudice, providing patients with resources for spiritual support, and helping patients fulfill their religious practices (Cinar & Arslan, 2017; Erol, 2020; Kokcu & Kutlu, 2020).

Spiritual care can help patients grasp the meaning of life, achieve inner peace, develop coping strategies that can help them overcome illness and crises, plan for the future, and accelerate the healing process (Ismailoglu et al., 2019). It was reported that spiritual care strengthens physical and mental health, supports disease self-management, increases individual satisfaction, and improves the quality of life (Chen et al., 2021; Cinar & Arslan, 2017; da Silva et al., 2009; Gergianaki et al., 2019; Kacal & Demirsoy, 2018; Kotli´nska-Lemieszek et al., 2022). Spiritual and psychosocial needs are more abstract and more complex than physical needs and are also difficult to measure. Therefore, physical needs, which can be measured more precisely and easily, are prioritized in the healthcare processes, whereas spiritual needs may be overlooked. Holistic and humanistic care requires identifying the spiritual needs of individuals and providing the necessary care (Benito et al., 2014). The spiritual care needs of patients with COPD are not sufficiently known (Hasegawa et al., 2017; Kotli´nska-Lemieszek, et al., 2022; Tzounis et al., 2016). This situation suggests that a holistic assessment has not been made.

Health professionals need to identify the physical, psycho-social, and spiritual needs of patients to provide quality and holistic care (Adugbire & Aziato, 2020; Hasegawa et al., 2017; Kotli ´nska-Lemieszek et al., 2022). However, health professionals often overlook the importance of spiritual care needs. Health professionals are often not able to properly address and incorporate spiritual care needs into clinical practice, either due to a lack of time or knowledge and skills (Gergianaki et al., 2019). In this regard, this study aimed to determine the relationship between spiritual care needs and death anxiety in individuals with COPD. The lack of a similar study in the literature increases the significance of the present study. The findings obtained in this study are expected to contribute to the literature and the patient care process of health professionals who provide treatment and care to patients with COPD.

## Method

### Purpose and Type of the Study

This descriptive and correlational study aimed to determine the need for spiritual care and death anxiety in patients with COPD and to identify the relationship between them. In this regard, the present study sought answers to the following questions:

- What is the level of spiritual care needs of patients with COPD?
- What is the level of death anxiety in patients with COPD?
- Are spiritual care needs and death anxiety related in patients with COPD?
- Is the need for spiritual care effective in death anxiety in patients with COPD?

### The Universe and the Study Sample

The universe of the study consisted of patients admitted to the pulmonology outpatient clinic of a public hospital between 1 November 2023 and 31 April 2024 due to the diagnosis of COPD. The sample size of the study was calculated as a minimum of 438 using the universe-known sampling method formula (Nt^2^pq/d^2^(N-1) + t^2^pq). Considering the possibility of loss, the sample size was increased by 10%, and a total of 480 patients were included in the study. A total of 32 patients could not participate in the study due to hearing and vision problems (n = 7), not volunteering for the study (n = 18), and not being able to speak due to the oxygen mask (n = 7). We adhered to the STROBE checklist for reporting.

### Inclusion Criteria

Being over 18 years of age, being diagnosed with COPD for at least one year, being able to communicate verbally, and being conscious.

### Exclusion Criteria

Refusal to participate in the study, cognitive or communicative inability to answer data forms.

### Data Collection Tools

The study data were collected using the Patient Diagnosis Form, Dyspnea Fear Level Assessment Form, Spiritual Care Needs Inventory, and Templer Death Anxiety Scale. The research data were collected by a researcher involved in the study. The data were collected by a single researcher. The research questions were collected by face-to-face interviews with the patients.

*Patient Diagnosis Form* The form was prepared by the researchers after reviewing the literature. This form consists of 17 questions that include socio-demographic characteristics of the patients (age, gender, marital status, education, employment status, etc.) and disease characteristics (duration of the disease, presence of additional chronic diseases, development of disease-related complications, etc.) (Arslan & Unsal, 2021; Asiret & Okatan, 2019).

*Dyspnea Fear Level Assessment Form* The Visual Analog Scale ranging from "0" (no fear due to dyspnea) to "10" (high level of fear due to dyspnea) was used to determine the fear levels of the patients due to dyspnea. In addition, patients were asked to categorize their level of fear as "none", "moderate", and "severe".

*Spiritual Care Needs Inventory* The scale developed by Wu et al., (2016) provides information about patients’ spiritual care needs. The scale consists of 21 items graded on a 5-point Likert scale. The scale consists of two sub-dimensions: "meaning and hope" and "caring and respect". Scores that can be obtained from the scale vary between 21 and 105. An increase in the overall mean score of the scale indicates a greater need for spiritual care. The Turkish validity and reliability study of the scale was conducted by Ismailoglu et al., (2019). The overall scale internal consistency Cronbach’s Alpha value of the Turkish version was calculated as 0.94 (Ismailoglu et al., 2019). In present study, Cronbach’s alpha value of the scale was calculated as 0.93, and it was determined to have a high level of reliability.

*Templer Death Anxiety Scale* The scale developed by Templer (1970) was first adapted into Turkish by Senol (1989). Akca and Kose (2008) revised the Turkish revision of the scale in different groups in Turkish norms and reconducted the validity and reliability study. The scale consists of 15 true–false dichotomous Likert-type items. Correct answers are given a "1" point, while incorrect answers are not included in the scoring. The score that can be obtained from the scale varies between 0 and 15. A higher score is interpreted as an increase in death anxiety. The average score on the scale is seven. People with a score of seven and above are considered to have high death anxiety. The reliability coefficient of the scale, for which a Turkish validity and reliability study was conducted, was found to be 0.75 (Akca & Kose, 2008). In present study, Cronbach’s alpha value of the scale was found to be 0.73, and it was determined to have an acceptable reliability.

## Data Analysis

The study data were analyzed using SSPS 22.0 software. The normality of the data was checked with the Kolmogorov–Smirnov test. The distribution of the individual and disease characteristics of the patients and the mean scale scores are shown by percentage and mean test. Pearson correlation analysis was used to determine the relationship between the mean scores of the Spiritual Care Needs Inventory and the Templer Death Anxiety Scale. Simple Linear Regression Analysis including a dummy variable was used to determine the explanatory effect of spiritual care needs on death anxiety. Student’s t-test was used to compare the age of the patients and the scale mean scores. Statistical significance level was taken as 0.05.

## Findings

The mean age of the patients was 69.76 ± 9.97 years and 71% were 65 years of age or older. Among the participants, 58.1% were male, 27.5% were illiterate, and 54.4% were primary school graduates. While 67.9% of the patients were married, 96.7% were not employed, and 63.7% considered their economic status to be good. Only 15% of the patients live alone. While 69.8% of the patients can meet their daily needs by themselves, 7.1% cannot meet their daily needs at all. The rate of current smokers was 16.7% (Table 1).

**Table 1:** Individual characteristics of patients

Characteristics	n	%
Age (years) (mean ± SD)	69.76 ± 9.97	
< 65	139	29.0
≥ 65	341	71.0
*Gender*		
Woman	201	41.9
Man	279	58.1
*Education level*		
Is illiterate	132	27.5
Primary school graduate	261	54.4
Secondary school graduate	63	13.1
High school graduate and above	24	5.0
*Marital status*		
Married	326	67.9
Single	154	32.1
*Working status*		
Yes	16	3.3
No	464	96.7
*Living alone situation*		
Yes	72	15.0
No	408	85.0
*Economic situation*		
Good	114	23.8
Middle	306	63.7
Bad	60	12.5
*Smoking status*		
Smokes	80	16.7
Quit smoking	220	45.8
Never smoked	180	37.5
*Ability to meet daily needs*		
Yes	335	69.8
Partially	111	23.1
No	34	7.1

While 43.1% of the patients had been diagnosed for 11 years or more, 83.5% of them received information about their disease from health professionals. The majority of the patients (81.9%) had other chronic diseases other than COPD, and the most common were hypertension (53.1%), diabetes (36.7%), and hyperlipidemia (15.2%). The level of fear of experiencing dyspnea due to the disease was found to be very high at a rate of 42.1% (Table 2).

**Table 2:** Disease characteristics of patients

Characteristics	n	%
*Disease duration*		
1–5 years	156	32.5
6–10 years	117	24.4
11 years and above	207	43.1
*Receiving education from a doctor or nurse about his/her disease*		
Yes	401	83.5
No	79	16.5
*Presence of another chronic disease other than COPD*		
Yes	393	81.9
No	87	18.1
Chronic diseases other than COPD*		
Hypertension	255	53.1
Diabetes	176	36.7
hyperlipidemia	73	15.2
Heart failure	70	14.6
Osteoarthritis	60	12.5
Other**	35	9.3
Level of fear of experiencing dyspnea (Mean ± SD)	6.18 ± 2.99	
No	115	24.0
Middle	163	34.0
Too much	202	42.1

*The number n has changed due to the number of individuals with more than one chronic disease

**Epilepsy, gastric ulcer, cancer

In this study, the mean Spiritual Care Needs Inventory was found to be 64.38 ± 17.92. Considering the range of scores obtained from the scale, the spiritual care needs of the patients were determined as moderate. It was found that patients had the highest need for spiritual care in the sub-dimension of caring and respect (27.95 ± 7.86). The mean score of the Templer Death Anxiety Scale was 7.70 ± 3.46 and 60.8% of the patients were found to have high death anxiety according to the scale cut-off value (Table 3).

**Table 3:** Distribution of patients’ mean scores on the spiritual care needs ınventory and templer death anxiety scale

Scales	Min–max points	Mean ± SD	n	%
Spiritual care needs ınventory	21–105	64.38 ± 17.92		
Meaning and hope	13–65	36.42 ± 11.58		
Caring and respect	8–40	27.95 ± 7.86		
Templer death anxiety scale	0–15	7.70 ± 3.46		
Death anxiety is low	< 7		188	39.2
Death anxiety is high	≥ 7		292	60.8

A weak, positive, and statistically significant correlation was found between the mean scores of the Spiritual Care Needs Inventory and the Templer Death Anxiety Scale (r = 0.327; *p* < 0.01). In this regard, it was determined that the spiritual care needs of patients with high death anxiety were higher in general and in all sub-dimensions (*p* < 0.01) (Table 4).

**Table 4:** The relationship of patients’ spiritual care needs with death anxiety

Spiritual care needs ınventory	Templer death anxiety scale			
	Total score test*, *p*	Death anxiety is low (n = 188)	Death anxiety is high (n = 292)	Test**, *p*
Meaning and hope	r = 0.333; *p* < 0.001	32.67 ± 11.77	38.84 ± 10.80	t = − 5.899; *p* < 0.001
Caring and respect	r = 0.255; *p* < 0.001	25.95 ± 8.07	29.23 ± 7.45	t = − 4.556; *p* < 0.001
Total	r = 0.327; *p* < 0.001	58.62 ± 18.38	68.08 ± 16.63	t = − 5.833; *p* < 0.001

*Pearson correlation analysis; **Studen’s t-test

As can be seen in Table 5, it was determined that gender, fear of experiencing dyspnea, and death anxiety predicted spiritual care needs at a level of 20% in patients with COPD (R = 0.457, R^2^ = 0.209, F = 17.800, *p* < 0.001). In addition, it was found that the need for spiritual care was higher in female patients (t = 6.162, *p* < 0.001).

**Table 5:** Regression analysis of death anxiety and spiritual care needs

Model	*B*	*SE*	*ß*	*t*	*p* value	95.0% confidence ınterval	
						Lower	Upper
*Spiritual care needs ınventory*							
Age	0.099	0.077	0.055	1.284	0.200	− 0.052	0.251
Gender	− 6.625	1.569	− 0.183	− 4.222	p < 0.001	− 9.709	− 3.541
Daily life aktivitelerini yapabilme durumu	1.959	1.310	0.067	1.496	0.135	− 0.615	4.534
Disease duration	− 0.724	0.884	− 0.035	− 0.819	0.413	− 2.462	1.014
Receiving information about the disease	− 0.200	2.033	− 0.004	− 0.098	0.922	− 4.194	3.795
Fear of experiencing dyspnea	1.343	0.265	0.224	5.072	p < 0.001	0.823	1.863
Death anxiety	1.094	0.229	0.211	4.786	p < 0.001	0.645	1.543

R = 0.457, R^2^ = 0.209, F = 17.800, *p* < 0.001

## Discussıon

The human being is a multifaceted being with bio-psycho-socio-cultural and spiritual dimensions, and these aspects have a correlation and influence on each other (Cinar & Arslan, 2017). Diseases that require long-term treatment increase the spiritual needs of patients in addition to their physical, emotional, mental, and social needs (Ismailoglu et al., 2019). In this regard, the present study examined spiritual care needs, death anxiety levels, and the correlation between spiritual care needs and death anxiety levels of patients with COPD.

The present study concluded that the spiritual care needs of the patients were at a moderate level (Table 3). In a previous study conducted with elderly individuals with COPD, the level of patients’ spiritual care needs was moderate (Chen et al., 2021). In studies conducted with patients hospitalized in the clinic before orthopedic surgery and with ostomy patients, it was determined that the spiritual care needs of the participants were at a moderate level (Alcan et al., 2022; Ayik et al., 2021). In a study conducted with hospitalized patients, it was reported that patients have spiritual needs that need to be met during their hospital stay (Nixon & Narayanasamy, 2010). A previous qualitative study determined that the spiritual needs of patients with COPD were not adequately met in the clinical setting. In contrast, it was stated that spirituality can be the "breath of God" that helps patients’ life questions and perspective on life (Tzounis et al., 2016). The present study was carried out in Turkey, a country where Islam is widely practiced. In the Islamic faith, the idea that both health and illness come from Allah is dominant, and the desire for spiritual power and Allah’s help in the fight against illness comes to the fore. Therefore, it can be stated that this belief is also dominant in individuals with COPD within the scope of the present study, they have difficulties in their struggle with their diseases, and their spiritual care needs increase.

Recognizing and considering the spiritual care needs of patients by health professionals improves the quality of care (Ustundag et al., 2024). The present study determined that patients mostly needed spiritual care in the sub-dimension of caring and respect (Table 3). Similar results were obtained in a study examining the preoperative spiritual needs of orthopedic surgery patients (Alcan et al., 2022). Cheng et al., (2018) found that cancer patients have a high need for hope and peace in spiritual care. The literature and the results of the present study are consistent. These results are noteworthy in terms of demonstrating that patients who have been traumatized by difficult disease processes and who need professional support need to be respected and cared for more than ever as an ethical and human right.

Physical health problems can cause death anxiety in individuals with chronic diseases (Okur & Nural, 2022). The present study determined that more than half (60.8%) of the patients with COPD experienced high levels of death anxiety (Table 3). Parallel to the present study, a previous study conducted with elderly patients with COPD found that 75.5% of the participants experienced death anxiety (Nal et al., 2016). Demir Gokmen and Firat (2022) found that death anxiety in patients with COPD was at a moderate level. In the same study, it was determined that the majority of patients with COPD (68.5%) perceived death as fate or punishment and felt uncomfortable being asked questions about death. Another qualitative study determined that all patients expressed fear of death (Banzett et al., 2020). Unsal and Yetkin (2005) found that 73% of patients with COPD thought about death more after the disease, 28.6% desired to die, and 13.9% experienced death anxiety.

Death anxiety is considered significant because it affects the individual negatively in psycho-social terms and causes difficulties in adapting to life and the environment (Turan Kavradim et al., 2022). In this regard, the findings of the present study reveal that death anxiety is experienced intensely in patients with COPD. We associate this finding with the fact that chronic illness significantly complicates activities of daily living, as well as with the characteristics of the patients in the sample, such as advanced age, long duration of disease, and frequency and severity of challenging symptoms.

Meeting spiritual needs helps the individual accept the disease and plan for the future, thus positively affecting the recovery process and increasing life expectancy (Cinar & Arslan, 2017). In this regard, the present study determined that death anxiety may decrease as patients’ spiritual care needs are met (Table 4). In addition, it was observed that patients with high death anxiety had higher spiritual care needs. A previous study determined that spirituality was positively associated with resilience and self-management in patients with COPD (Chen et al., 2021). Mendes et al., (2021) reported that patients with COPD with high spiritual health showed less tendency to anxiety and depression, as well as better emotional functioning and disease control. Another study revealed a positive correlation between spiritual health and medication adherence in patients with COPD (Helvaci et al., 2020).

In addition to death anxiety, patients with COPD may face various negative factors such as impairment of body integrity, future anxiety, separation from their relatives and familiar environment, pain and invasive interventions, life being under threat, being bedridden, disruption of sleep patterns, dependence on devices or intensive care unit, and not being adequately informed about the disease, treatment, and practices (Kacal & Demirsoy, 2018). Therefore, it is predicted that by supporting and strengthening the spiritual aspect of patients with COPD, disease management can be improved. In this regard, significant advancement can be achieved in reducing symptoms and improving quality of life, as well as reducing death anxiety.

The present study determined that female gender, fear of experiencing dyspnea, and death anxiety variables increased the need for spiritual care by 20% in patients with COPD (Table 5). Similar to the present study, Hasegawa et al., (2017) reported that dyspnea severity significantly affected spiritual health in patients with COPD. In particular, patients with COPD experience respiratory symptoms, which is an important vital parameter. An increase in the severity of dyspnea can cause patients to death due to conditions such as an inability to breathe and asphyxiation. This experience may increase death anxiety due to the fear of dyspnea in patients. In such cases, many patients may resort to spirituality to manage their condition. In this regard, our study concluded that the spiritual needs of patients with dyspnea fear increased.

The present study determined that the need for spiritual care was higher in female patients (Table 5). Similarly, it was determined that spirituality (Julianna & Koronczai, 2021) and positive religious coping levels (Nascimento et al., 2019) were higher in females than males. Galek et al., (2008) found that females experience spiritual needs such as belonging, meaning, hope, beauty, and acceptance of death much more frequently than males. However, spiritual needs related to morality concerns or religious practices do not differ according to gender. In this regard, we associate this finding with the differences between male and female gender in terms of physical, emotional, social, and cultural characteristics.

### Limitations of the Study

This study has several limitations. First, the study was conducted with only COPD patients admitted to a single public hospital during a specific time period. The data on spiritual care needs and death anxiety of the patients in the study are limited to the scales used and are based on the self-reports of the patients. It does not provide information about what spiritual care needs patients have. In addition, since not every patient had a respiratory function test result, disease stage and spiritual need and fear of death according to stage were not evaluated.

## Conclusion

The present study found that Turkish patients with COPD had moderate level spiritual care needs, more than half of the patients had high levels of death anxiety, and by meeting spiritual care needs, death anxiety may be decreased. Within the framework of the holistic care approach, questioning the spiritual care needs of patients should be the first and most essential approach of health professionals. Additionally, spiritual care should be integrated into holistic patient care. In addition, experimental studies assessing the effect of nursing interventions regarding the spiritual care needs of patients on death anxiety are recommended. Similarly, longitudinal studies to monitor changes in spiritual care needs and death anxiety over time or intervention studies to assess the impact of spiritual care on these outcomes are recommended for future research.
